# Voltine Ecotypes of the Asian Corn Borer and Their Response to Climate Warming

**DOI:** 10.3390/insects12030232

**Published:** 2021-03-09

**Authors:** Kai-Qiang Liu, Lian-Xia Wang, Tian-Tao Zhang, Shu-Xiong Bai, Ke-Qin Wang, Zhen-Ying Wang, Kang-Lai He, William D. Hutchison

**Affiliations:** 1 The State Key Laboratory for Biology of Plant Diseases and Insect Pests, Institute of Plant Protection, Chinese Academy of Agricultural Sciences, Beijing 100193, China; liukaiqiang0625@163.com (K.-Q.L.); zhtiantao@163.com (T.-T.Z.); sxbai@ippcaas.cn (S.-X.B.); zywang@ippcaas.cn (Z.-Y.W.); 2 Qiqihar Sub-Academy of Heilongjiang Academy of Agricultural Sciences, Qiqihar 161006, China; wlx0427@163.com; 3 Institute of Plant Protection, Heilongjiang Academy of Agricultural Sciences, Harbin 150000, China; Wang.keqin@163.com; 4 Department of Entomology, University of Minnesota, St. Paul, MN 55108, USA; hutch002@umn.edu

**Keywords:** *Ostrinia furnacalis*, climate change, voltinism, ecotype, diapause, photoperiod

## Abstract

**Simple Summary:**

The Asian corn borer (ACB), *Ostrinia furnacalis*, is an important economic pest of corn and other crops, and is widely distributed in China. Depending on the climate, ACB may have one or up to seven generations each year, from north to south, respectively. The degree of crop damage is closely related to its phenology and number of generations per year (voltinism). Climate warming may result in an alternation of voltinism in the ACB. In the present study, we investigated the voltinism of different populations under different simulated environments. From the diapause response, both the uni- and multivoltine ecotypes were coexisting in the Harbin (H) population collected from action sites of ACB moths in Harbin, Heilongjiang Province, China. Diapause incidence declined given the climate-warming scenario, which was temporally specific and could be overridden by significantly low daily average temperatures. Elevated CO_2_ did not directly impact voltinism. On the basis of voltinism, the H population reflected sympatric uni- and multivoltine ecotypes, with multivoltinism being dominant. The univoltinism trait was recessive. Climate warming could significantly override photoperiod effects. Warmer temperatures and declining latitude (reduced daylength), and their interaction, are estimated to drive ACB evolution to increased homogeneity and multivoltinism.

**Abstract:**

In the Asian corn borer (ACB), *Ostrinia furnacalis* (Guenée), diapause is governed by a multigenetic constitution that responds to daylength and temperature with seasonality. The ACB displays uni- or multivoltinism, depending on its geographic specificity. Hence, warmer temperatures may result in alternation of voltinism in the ACB, which will help in understanding the ecological consequences of climate warming on insects. In the present study, we investigated the voltinism in two natural populations from Harbin (H) and Gongzhuling (G) as well as a laboratory (L) population (established from the H population in 2017) of the ACB, at ambient and elevated atmospheric CO_2_ (*a*CO_2_ 390 μL/L and *e*CO_2_ 750 μL/L) and temperature (*a*T and *E*t = *A*t + 2 °C). From the diapause response, both the uni- and multivoltine ecotypes were coexisting in the H population. The neonate occurrence date of 50% individuals that induced diapause was ca. 10 days later in the G population than in the H population, but it was about 10 days earlier than in the L population. Comparing to the dates of onset and the peak of diapause induction, the G and L populations were less variable than the H population in response to a short and/or shortening daylength in the field. The univoltine individuals could not be eliminated completely after 19 generations of selection. Diapause incidence decreased with a climate-warming scenario, which was temporally specific and could be overridden by significantly low daily average temperatures. The *e*CO_2_ did not directly impact the voltinism. On the basis of voltinism, the H population was sympatric for uni- and multivoltine ecotypes, with multivoltinism being dominant. The univoltinism trait was recessive. Climate warming could significantly override the effect of photoperiod, which was yearly dependent. Warmer temperatures and a decreased latitude (shortened daylength), and their interaction, would drive ACB evolution toward diapause homogeneity for multivoltinism.

## 1. Introduction

Diapause is a highly adaptive trait that enables herbivorous insects to adapt to a tremendous diversity of climatic conditions, and thus harmonize phenology with seasonal host plant availability in temperate regions. Depending on the degree to which diapause is governed by environmental cues, diapause strategies are often distinguished as facultative and obligatory [1,2]. Insects with obligatory diapause will only exhibit one generation and enter diapause at a certain stage during development regardless of the photoperiod or temperature. In this case, the diapause is genetically preprogrammed. By contrast, insects with facultative diapause are induced by environmental factors, and usually exhibit multivoltinism along with an expansive geographical distribution. Voltinism plasticity is dependent on latitude, including the onset of diapause, which is induced by photoperiod. Therefore, voltine ecotypes have often evolved a unique diapause response and become synchronized seasonally with their ecotypes. Although the voltine ecotypes are virtually identical in morphology, both genetic and molecular differences have been documented in several insect species, such as the European corn borer, *Ostrinia nubilalis* (Hübner) [3,4].

Although daylength is the major environmental cue regulating the onset of diapause in insects with a facultative diapause, temperature can alter the diapause response to the daylength [5]. In particular, the compensatory effect of temperature has been reported in many insects. The flesh-fly, *Sarcohpaga argyrostoma* (Robineau-Desvoidy), is a long-day species with 13.5–14 h light/24 h as a critical daylength at both 15 and 20 °C; however, diapause is hardly induced at 25 °C [6]. An upward shift in temperature, such as 5 °C, could significantly eliminate larval diapause incidence in response to a short day at 23 °C in the yellow peach moth, *Conogethes punctiferalis* (Guenée) [7]. In some insects, diapause induction is a temperature-dependent process, and the effect of photoperiod is only demonstrable at certain temperatures. For example, 25 °C is critical for *Diatraea grandiosella* (Dyar) [8].

As ectotherms, the physiological aspect of insects, such as metabolic rate, is dependent upon the environmental temperature. All salient biological parameters, such as developmental rate, are primarily driven by the temperature. Thus, higher ambient temperatures, including the impact of global climate change, can lead to rapid development, with the consequence that insects with a facultative diapause may be capable of increased voltinism [9,10,11,12,13,14].

The Asian corn borer (ACB), *Ostrinia furnacalis*, is endemic to Asia and the western Pacific region. In China, the pest’s distribution extends from the far northern cool temperate region to the southern tropical region, where the population displays phenotypic plasticity in voltinism. It can reproduce throughout the year with continuous overlapping generations in subtropical and tropical regions, where it can complete seven or more generations per year [15,16]. ACB egg masses can be found on sweet corn throughout the year with a single peak from early to mid-June in those regions [17]. The proportion of diapausing larvae can be detected every month, which is correlated to the mean low temperature of each month [18]. In temperate regions, ACB populations occur less abundant but produce more synchronized generations each year. Fully fed 5th instars may enter diapause at any generation [19]. Induction of facultative diapause is a predominant response to changes in daylength with a complementary effect of temperature [20]. The critical daylength increases with an increasing degree in latitude [20,21,22]. Critical daylength is variable between sympatric uni- and bivoltine ecotypes [23]. The differences among critical daylengths between sympatric uni- and bivoltine ecotypes are longer under alternating temperatures of 29/22 °C (L/D) (1 h 15 min) than under a constant temperature of 26 °C (47 min).

In the cool temperate regions of Heilongjiang and Jilin Provinces, ACB populations undergo 1 to 2 generations per year [24]. Recent research suggests the onset of altered voltinism has been changing in the Jilin Province because of climate warming. In the eastern, central, and western ecoregions of Jilin Province, voltinism has ranged from 1, 1.5, and 2 generations per year before 1980, respectively, to 1 and 1.5, 2 and 2.5, and 2 and 2.5 generations per year in 2012. This outcome is based on the egg-lay dynamics observed in the field (1 and/or 2 peaks) and immature larvae detected at the end of September [25].

**Figure 1:**
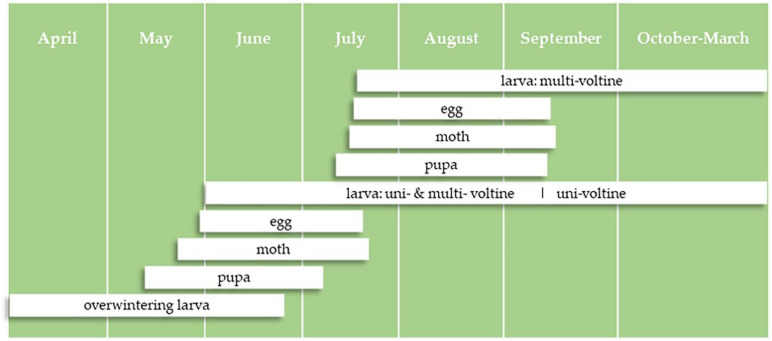
Typical life history and phenology of the Asian corn borer in calendar months.

ACB populations usually demonstrate distinguishable, synchronized generation peaks, where each generational moth flight lasts for ~40 days (Figure 1) [26]. The developmental time of overwintering larvae of the bivoltine ecotype (28.3 d at 26 °C) is shorter than that of the univoltine ecotype (48.5 d at 26 °C), which suggests that the bivoltine ecotype emergence occurs earlier in spring, with advanced phenology of reproduction and development [27,28]. Historically, variation in post-diapause developmental time between voltine ecotypes facilitated asynchrony in the mating period for ACB, thus minimizing genetic exchange between ecotypes [28]. This phenomenon also occurs in *O. nubilalis* [29,30,31]. However, given global climate change projections, we hypothesized that sympatric uni- and multivoltine ecotypes of ACB might respond differently to climate-warming scenarios in China. Therefore, the aim of this paper is to improve our ability to forecast the occurrence and contributions of ACB voltinism to population structure under the context of global warming.

## 2. Materials and Methods

### 2.1. Growth Conditions and Maize Variety

Octagonal open-top chambers (OTCs) [32] were used to simulate (1) elevated atmospheric CO_2_ (*e*CO_2_, 750 μL/L, predicted levels of CO_2_ in end of this century) [33] and elevated temperature (*e*T); and (2) ambient atmospheric (*a*CO_2_, 390 μL/L) and *e*T. Briefly, an OTC consists of 3 parts: (1) a chamber, which is made of an aluminum frame, transparent glass, standing on a regular octagonal wall (50 cm thick, 85 cm high, and 40 cm above ground) and cement base. The cement base is hollow inside with 16 vents for air-outlets, opening to inside the chamber, and two air-intake pipes from the base connecting to the gas supplier; (2) a CO_2_ and cool air supplier unit, which consists of air cooling systems, air fans, and CO_2_ tanks; and (3) a digital control system, which includes a CO_2_ sensor, 2 temperature and humidity sensors, a chip core, a computer, and a software package. When processing, digital controls will detect and analyze the CO_2_ and temperature levels, via the sensors and computer, and provide feedback signals to the CO_2_ and cool air supply unit (Figures S1 and S2). Field-based square screen cages (6 × 6 × 3 m^3^) were used as a control for ambient temperature (*a*T) and atmospheric CO_2_ (*a*CO_2_), which were about 1 km away from the OTCs (Figure S2). *e*T was ca. 2 °C higher than *a*T. In total, three climate regimes (*e*CO_2_ and *e*T, *a*CO_2_ and *e*T, and *a*CO_2_ and *a*T) were established for our experiments.

A single-cross hybrid corn (*Zea mays* L.), Jidan 209, was planted at several time intervals to ensure that the corn was in similar developmental stages when the plants were infested with ACB neonates. This hybrid is typical of those grown for commercial production, and the field management was consistent with the common cultural practices used in local farming.

### 2.2. Insects

Two natural populations (H and G) and a laboratory population (L) of ACB were used in this study. ACB moths were collected with nets in the weed/grass areas (“action sites for moths”) near each corn field in Harbin (45°38′ N, 126°34′ E), Heilongjiang Province, from 2017 to 2019, and in Gongzhuling (43°30′ N, 124°47′ E), Jilin Province, from 2018 to 2019; collections represented the H and G populations, respectively. In each year, we collected the ACB adults in both places from the middle of June to the end of July for 40 days. Moths collected about every 5 days (time period) were pooled according to the time sequence, respectively. We collected 40–333 females at each location in Gongzhuling or Harbin for each time period (Tables S1 and S2). The moths were brought to the laboratory and placed in an oviposition cage as described by Zhou et al. [34]. A piece of wax paper was placed on the top of the cage as a substrate for egg laying. Egg masses were harvested each day by collecting the wax paper, with immediate replacement for the subsequent daily oviposition. According to the experimental requirements, 150 to 600 egg masses (80–120 eggs per egg mass) per location, for each time period, were pooled and incubated to obtain a sufficient number of neonates for infestation, or laboratory rearing experiments.

An L population was designated as the laboratory colony that was originally collected from Harbin in the summer of 2017, and then maintained on ACB artificial diet in an environmental chamber at 28 ± 0.5 °C, 60–70% relative humidity, and a photoperiod of 16:8 h (L:D), for successive generations. In practice, larvae that are multivoltine develop to pupate within 15 to 20 days under these rearing conditions. Otherwise, the larvae are univoltine and in diapause (obligatory diapause). Larvae that pupated within 15 days under these rearing conditions would be maintained for the next generation. Diapause incidence was periodically assessed for a total of 464–3688 individuals with 2–4 samples per generation, i.e., the larvae would be reared for 45 days (avoiding any rare individual pupated after 20 days) under these conditions and then counted the pupae and larvae, respectively. After 18 generations, we assessed diapause incidence when larvae were reared at 28 °C and 60–70% RH with a photoperiod of 13:11 h (L:D). The L population was maintained for 8–9 generations in 2018 and 18–19 generations in 2019, when it was used for field infestations during each year.

### 2.3. Plant Infestation

Egg masses of the H and the G populations were incubated in environmental chambers at 28 °C, 60–70% relative humidity, and a photoperiod of 16:8 h (L:D) until hatching. Corn plants in the OTCs and field screen cages were infested with newly hatched larvae of three populations, respectively. Each plant was infested with 50 neonates (<12 h) of the ACB with traditional artificial infestation techniques described by He et al. [35]. Twenty-five plants were infested in each OTC or field screen cage. To avoid exposing the neonates to high temperature and direct sunlight, the infestations were done during the late afternoon to evening.

### 2.4. Laboratory Rearing

Apart from plant infestation, the neonates of the H population were also reared with the ACB artificial diet in the environmental chambers at 28 °C, 60–70% relative humidity, and a photoperiod of 16:8 h (L:D). The number of larvae and pupae were recorded separately after 45 days. If a larva developed to pupate within 45 days under these rearing conditions, it was deemed multivoltine, otherwise it was univoltine and in diapause (obligatory diapause).

### 2.5. Voltinism Assessment

Maize plants were dissected 45 days after infestation, which would ensure all larvae of the multivoltine individuals had completed development to pupation. The number of larvae and pupae were recorded separately. Diapausing larvae were counted as univoltine individuals whereas pupae were counted as multivoltine individuals.

### 2.6. Effective Accumulative Temperature Calculation

Temperature data for Gongzhuling and Harbin, from 2011–2019, were obtained from the website (http://tianqi.2345.com/wea_history/) (accessed on 20 August 2020). A degree-day calculator developed by the University of California (http://ipm.ucanr.edu/WEATHER/index.html) (accessed on 20 August 2020) was selected using the single sine-wave method with a horizontal cut-off to calculate effective heat unit accumulations. The lower (or base) threshold for development, and degree-days for ACB life stages, were for the egg stage 13.2 °C and 44.7; for the larval stage 6 °C and 452.1; and for the pupal stage 11.8 °C and 115.5 [36].

### 2.7. Experimental Design and Data Analysis

In total, three experiments were conducted in the study. The first experiment included two environmental conditions (i.e., laboratory-reared field screen cage conditions) × 8 time-interval sub-populations of the H population, as a factorial experiment with three replicates each (two replicates of field cage condition in 2019). The laboratory rearing condition was at 28 °C, 16:8 h (L:D) photoperiod, and 70% relative humidity from 2017 to 2019. The field screen cage condition was conducted from 2017–2019.

The second experiment was a 3 (ACB populations, i.e., H, G, and L) × 8 (time-interval sub-populations of each population) factorial experiment with a complete randomized design, with three replicates across two years (2018 to 2019) (two replicates of G and L populations in 2018, H population in 2019). The experiment was conducted in a field screen cage. Besides the H and the G populations that were collected in the field each year, the L population used in 2018 and 2019 was consecutively reproduced for 8–9 and 18–19 generations in the laboratory, respectively.

The third experiment was a 3 (environmental conditions) × 8 (time-interval sub-populations of the H population) factorial experiment with randomized design across three years (2017 to 2019). The experiment was conducted in OTCs and field screen cages, which simulated three environmental conditions, i.e., *e*CO_2_ and *e*T, *a*CO_2_ and *e*T, and *a*CO_2_ and *a*T. There were two replicates for treatments of *e*CO_2_ and *e*T and *a*CO_2_ and *e*T and three replicates for treatment of *a*CO_2_ and *a*T (two replicates in 2019). Each year, eight time-interval populations of the H population were infested into the corn plants growing in three environmental conditions from 2017 to 2019.

Data from Experiment 1 were classified into two datasets for each year on the basis of environments. Each dataset was subjected to a one-way ANOVA for each year with time (sub-population) as a single factor. Data from Experiment 2 were classified into eight datasets for each year on the basis of time (subpopulation). Each dataset was subjected to a one-way ANOVA for each year with population as a single factor. Two-way ANOVA was used to evaluate the effects of two different environmental factors (CO_2_ and T) in Experiment 3. Differences among treatment means were compared using Tukey’s HSD test (*p* = 0.05). Prior to analysis, the percentage data were subjected to standard transformations to improve their normality and the homogeneity of variance. For a given dataset, if all data were within the range of 30 to 70%, no transformation was done; if all data were within the range of either 0 to 30% or 70 to 100%, but not both, the square root was used; if all data did not follow the ranges specified here before, the arc sine transformation was used.

## 3. Results

### 3.1. Diapause Incidence of the H Population in the Laboratory and in the Field

**Figure 2:**
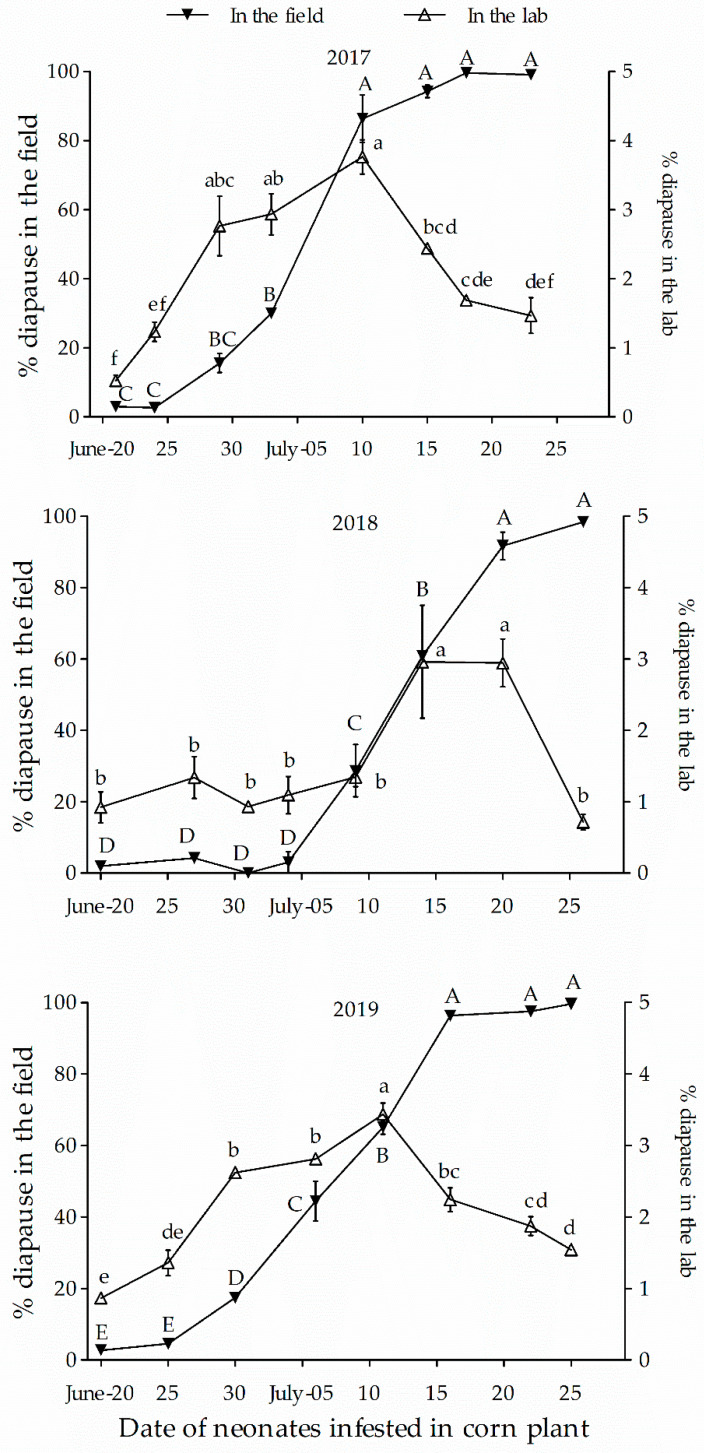
Dynamics of diapause incidence of the Harbin (H) population of *Ostrinia furnacalis* in the field and laboratory along with neonate occurrence in spring. Symbol and bars represent the mean ± SE values. Different uppercase and lowercase letters indicate significant differences (*p* < 0.05) among diapause incidence of the H population in the field and in the laboratory, respectively.

**Figure 3:**
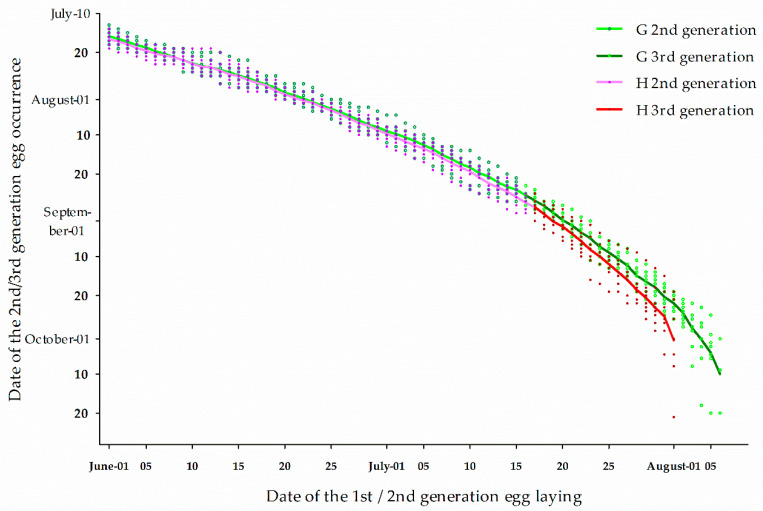
Predicted date of 2nd and 3rd generation egg occurrence of *Ostrinia furnacalis* based on the accumulative temperature requirement from the 1st/2nd generation egg occurrence in Gongzhuling and Harbin.

In the laboratory, when larvae were reared at 28 °C with a long day (L: 16 h), larval diapause incidence was generally very low during the time of neonate occurrence of the H population in spring, indicating a typical long-day response species (diapause only at short photoperiods) (Figure 2). Diapause incidence, meanwhile, was slowly increasing, but there was a significant increase by day that would peak on 10 July 2017, 15 July 2018, and 12 July 2019 (Figure 2, Table 1), respectively. After these dates, diapause incidence began to decline, although it was very low (peak value was less than 5%). In the field, the diapause incidence was low and did not fluctuate significantly in earlier hatched larvae of the population (end of June in 2017 and 2019, before 10 July 2018). However, the diapause incidence increased significantly after June and July (Figure 2, Table 1). The data indicate co-existence of uni- and multivoltine ecotypes in the population structure of overwintering larvae. In addition, multivoltine ecotype individuals exhibited earlier spring occurrence than the univoltine ecotype. The 2nd flight of multivoltine moths could occur from July 15th each year (Figure 3). On this account, the average incidence of multivoltine individuals ranged from 73.9–83.6% and 97.8–98.5% for the 1st flight population across six time periods (20 June to 15 July), under field and laboratory conditions, respectively.

### 3.2. Variation in Diapause Incidence Dynamics between Geographically Distinct Populations in the Field

**Figure 4:**
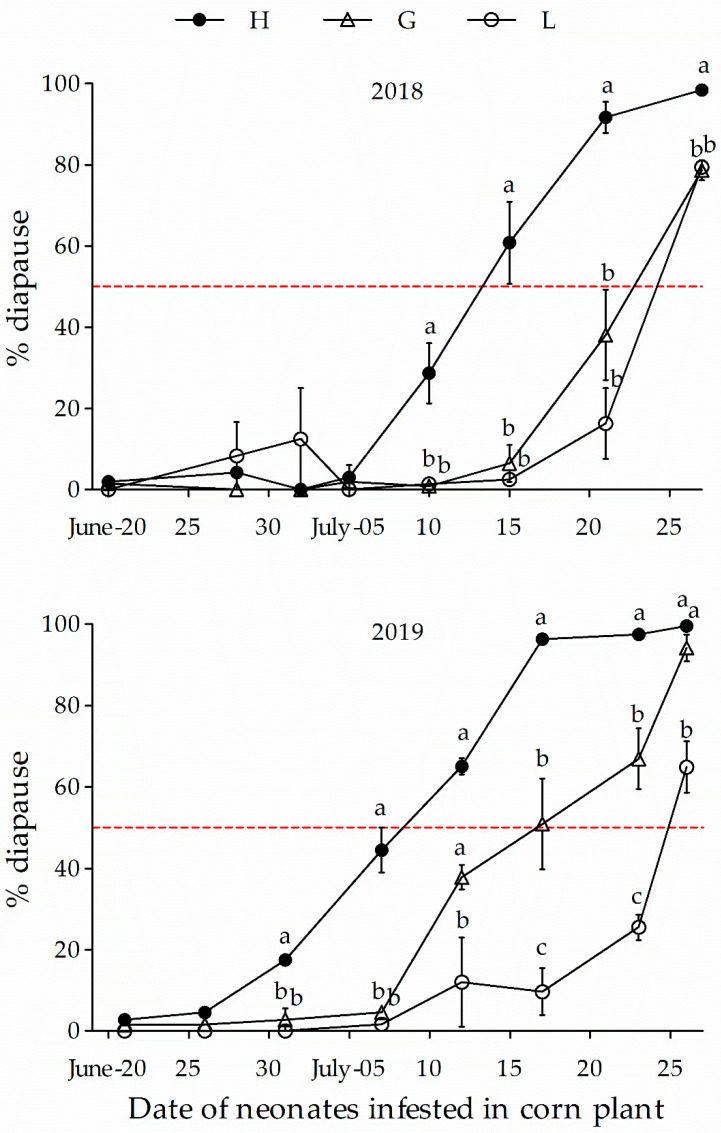
Percentage diapause of the Harbin (H), Gongzhuling (G), and laboratory (L) populations of *Ostrinia furnacalis* in the field, with egg hatching at different dates. Symbol and bars represent the mean ± SE values. Different lowercase letters for the same dates indicate significant differences (*p* < 0.05) in diapause incidence among populations.

In both the 2018 and 2019 experiments, the dynamics of diapause incidence exhibited a similar trend for all three populations, i.e., the diapause incidence was very low in June and had gradually increased by early to mid-July (Figure 4). By comparison, the onset of significant diapause induction was about 10 to 20 days earlier in the H population versus the G and/or L populations. In addition, the diapause incidence was significantly low in the L population over time in comparison with both the H and G populations (Figure 4, Table 2). Diapause was only significantly inducted by late July in the L population, which was about 7–10 days later than in the G population. However, onset of the highest percentage diapause occurred in the individuals hatching from around July 26th for all three H, G and L populations. Thus, the G and L populations had lower variation in response to short and/or shortening daylength (Figure S3), undergoing diapause more synchronously than the H population. The data indicate that the ACB is approaching more homogeneity for multivoltinism in an evolutionary context, driven by high temperature and/or short daylength, as well as their interaction.

### 3.3. Climate Warming and Diapause Induction

**Figure 5:**
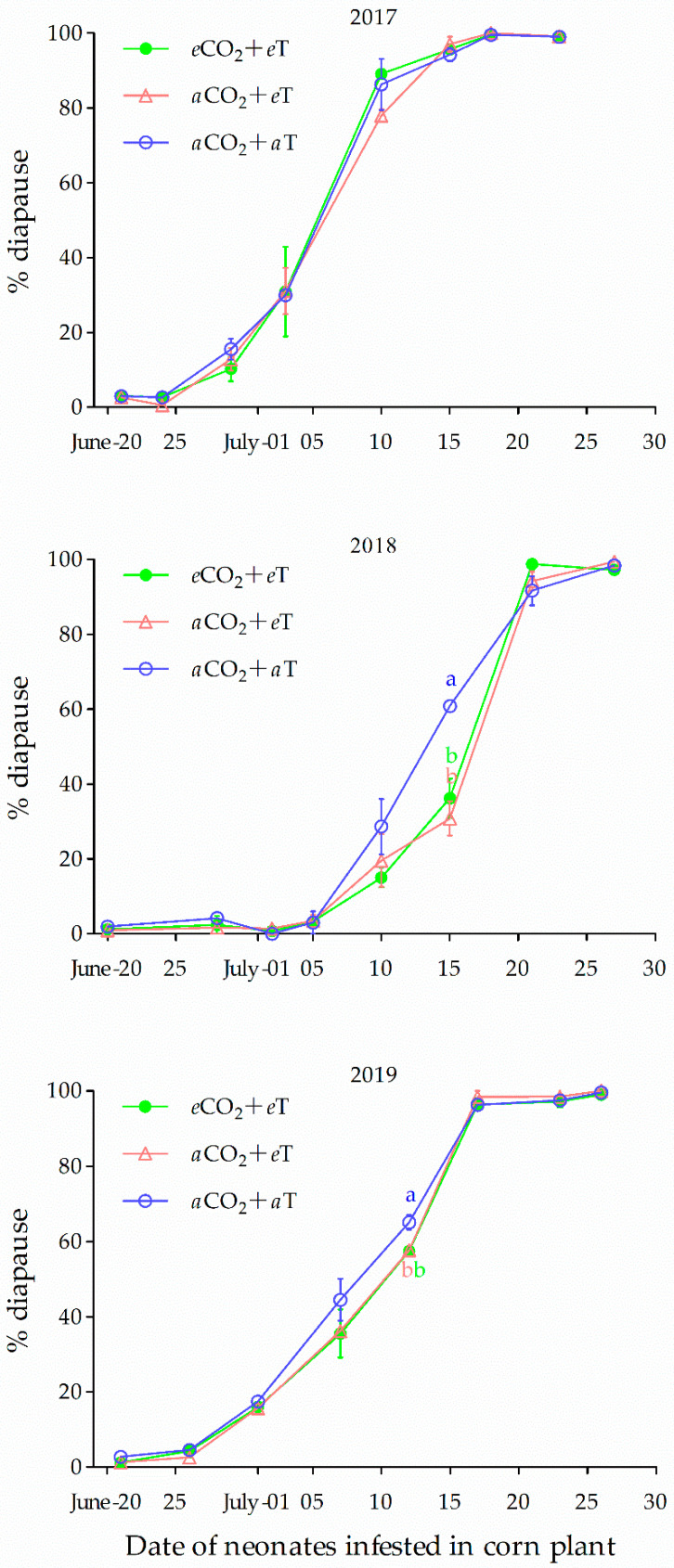
Diapause incidence of the H population of *Ostrinia furnacalis* developed in ambient and elevated CO_2_ and temperate environments. Symbol and bars represent the mean ± SE values. Different lowercase letters in the same date indicate significant differences between different environments in diapause incidence.

**Figure 6:**
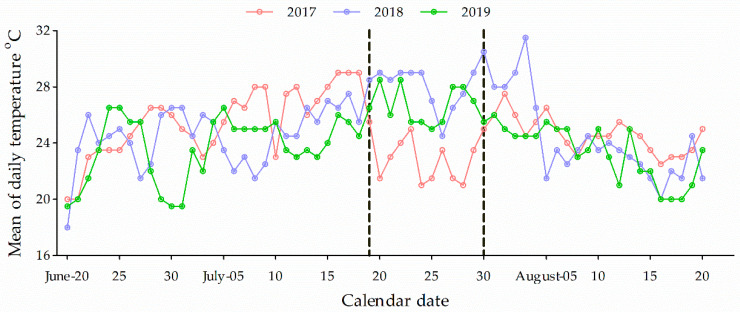
Average daily temperature in Gongzhuling, Jilin Province, China.

Regardless of the simulated environment, diapause induction of the ACB revealed a similar trend; the proportion of the larvae in diapause reflected an S-shaped logistic curve over time, for all 3 years (Figure 5). Overall, diapause incidence in response to environmental cues was exceedingly low for neonate occurrence before July (2017 and 2019 or 5 July in 2018). Soon after this time, diapause incidence rapidly increased until mid-July in 2017 and 2019; by 21 July in 2018, the ACB reached >90% diapause. Afterward, diapause incidence increased more slowly. The prediction that climate warming would decrease diapause incidence (increase the proportion of multivoltine individuals) was temporally specific. The onset of altered voltinism occurred significantly only around mid-July (i.e., emergence of neonates was from 7–15 July, Figure 5), and driven by *e*T. In addition, the effect of climate warming was also variable each year; i.e., significant effects were observed in 2018 and 2019 (*F*_1,5_ = 25.56, *p* = 0.0037; *F*_1,4_ = 43.47, *p* = 0.022), but not in 2017. Coincidently, the dynamics of daily mean temperature change during 19–30 July (for larval developmental time) were similar between 2018 and 2019, but exhibited a dramatic decline in 2017 (Figure 6). The *e*CO_2_ did not have significant impact on the voltinism of the ACB (*p* > 0.05).

### 3.4. Diapause Incidence of L Population

**Figure 7:**
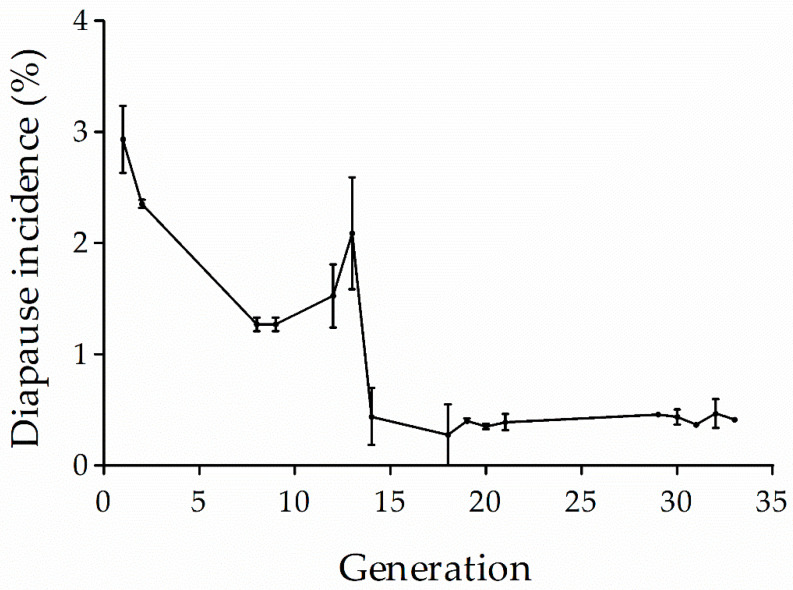
Diapause incidence of L population larvae reared at 28 °C with a photoperiod of 16:8 h (L:D). Symbol and bars represent the mean ± SE values.

Aperiodic estimates of diapause incidence throughout the laboratory rearing and across generation indicate that the L population consistently exhibited a very low incidence of diapause when larvae were reared at 28 °C with a photoperiod of 16:8 h (L:D) (Figure 7). Overall, the diapause incidence declined across generations although it was fluctuant. Importantly, there still were 0.3–0.5% of individuals entering diapause after 30 generations. After 18 generations, the diapause incidence was as high as 98.0 ± 0.8% when larvae were reared at 28 °C with a photoperiod of 13:11 h (L:D).

## 4. Discussion

A number of studies have shown that ACB is an insect with facultative diapause in response to short days in autumn, with a seasonal rhythm [20,21,22,23,37,38]. The temperature thresholds for egg, larval, and pupal development are 13.2, 6.0, and 11.8 °C, respectively, and the cumulative temperature requirement for one generation (from egg to egg) is ca. 612 degree-days (egg, 44.7; larva, 452.1; and pupa, 115.5) [36]. The cumulative temperature requirement for 2nd generation egg and larval development (from egg to full-fed larvae that enter diapause for over- wintering) is 497 degree-days. Corn is normally planted between late April to early May in Harbin, Heilongjiang Province. The 1st flight of the moths for the multivoltine ecotype, developing from overwintering larvae, usually occurs between early to late May. The resources available (accumulative temperature requirement, host plants) is sufficient to produce 2 or even 2.5 generations of the ACB in Harbin (Figure 3). Predicted adult emergence of the first generation (the 2nd flight) is approximately July 15. The present study revealed that the H population consists of a sympatric mix of multi- and univoltine ecotypes. However, at this location, the multivoltine ecotype was dominant, comprising 73.9–83.6% and 97.8–98.5% of the 1st flight population across six time periods (20 June to 15 July) in the field and in the laboratory conditions, respectively. Although a few single generation individuals (obligatory) directly entered diapause while their neonates had been hatching on June 20 (i.e., the earliest date for testing), onset of diapause induction might not occur until the multivoltine neonates had hatched and were developing from July 11–20 (Figure 2). These dates are also in agreement with the predicted dates for the onset of the 2nd flight. These dates are also earlier than the expected date (5th of August), where the critical photoperiod (15 h/43 min) for diapause induction occurs [39].

In the present study, the critical daylengths were shortening from 16 h/23 min and 16 h/8 min for the Harbin and Gongzhuling univoltine ecotypes in the field, respectively. In contrast to the H population, the critical daylength was significantly shorter for the L population (originated from Harbin), which declined to 15 h/56 min. In fact, to establish the L population, only individuals developed to pupate within 15 days were used for continuous regeneration in the laboratory, which would eliminate individuals with the diapause phenotype (univoltinism). In previous research, under constant temperatures, the critical daylengths for diapause induction (i.e., the daylength that induces 50% of individuals entering diapause) were 15 h/53 min, 15 h/43 min, and 15 h/8 min for the Harbin population, at 22, 25, and 28 °C, respectively [39]. The critical daylengths were 15 h/48 min and 14 h/53 min at alternating temperatures of 29/22 °C (L/D = 16/8 h) and at a constant temperature of 26 °C for the Gongzhuling univoltine ecotype [23]. Given our current results, the data indicate that multivoltinism was a dominant trait in the L population. Although selection had been conducted for 8–9 (2018) and 18–19 (2019) generations, a few univoltine individuals were observed whenever neonates were placed on corn in the field before 10 July. This suggests that the univoltinism (obligatory diapause) trait was persistently recessive in the H population. Similar results have been reported for *O. nubilalis* in the U.S. [40,41].

A recent study reported that the ACB’s geographical populations fit a latitudinal cline [16]. Hybrid offspring from crossing with northern geographic strains, such as Harbin, and with southern strains, such as Ledong (Hainan Province), normally showed an intermediate critical daylength [39,42,43]. It is worth noting that the range of daylength that individuals respond to to enter diapause is broader in hybrids (F1, F2, and backcrosses) than in their parents. In the present study, on the basis of significant diapause induction, the H population showed the broadest range from the date of onset to the date of peak diapause incidence, followed by the G population, and the L population was the shortest. Therefore, the H population should have a broad genetic background (heterogeneity) for multivoltinism and the L population had evolved more homogeneity regarding multivoltinism, suggesting that the evolution of ACB tends towards homozygosity of the multivoltine ecotype, driven by high temperatures and a short daylength, and their interaction.

As a result of climate warming, multivoltine insects are able to gradually increase as a proportion of the total population, rather than experience a complete switch in voltinism [44]. The influence of a short day length induced diapause in ACB, which could be modified by temperature [16,20]. Results of the present study indicate that higher temperatures could inhibit diapause induction of individuals whose neonates hatched during 7 to 17 July 2018 and 2019. However, this effect of climate warming could be averted if the temperature was lower than normal during critical periods of the season. For example, daily mean temperatures were significantly lower during 19 to 30 July 2017, than that in 2018 and 2019 (Figure 6). The unusually lower temperatures during this period resulted in a diminishing effect of higher temperatures facilitated by climate warming, such that there was no significant difference in larval diapause incidence among simulated climate warming and ambient conditions in the field during 2017. Of critical importance, given the primary outcome of increasing multivoltinism in the ACB for several climate-warming scenarios, the risk of feeding damage and maize yield loss in China is likely to increase [16,24]. Going forward, Integrated Pest Management (IPM) strategies may also need to change for the ACB, as has been the case for *O. nubilalis* in North America [45,46].

## 5. Conclusions

Diapause incidence in ACB decreased for the climate-warming scenario (*e*T = *a*T + 2 °C), which was temporally specific. However, diapause incidence was also influenced significantly by ambient temperature, specifically low daily average temperatures. On the basis of voltinism, the H population was characterized by sympatric uni- and multivoltine ecotypes, with multivoltinism being dominant. The univoltinism trait was recessive and informed by quantitative genetics. Climate warming could significantly override the influence of photoperiod. Warmer temperatures, resulting from climate warming and a declining latitude (shortened daylength), and their interaction, could drive ACB evolution to more homogeneity in multivoltinism.

## Figures and Tables

**Table 1:** One-way ANOVA statistics for each year with time as a single factor.

Parameters	Laboratory			Field Screen Cage		
	2017	2018	2019	2017	2018	2019
df	7, 16	7, 16	7, 16	7, 16	7, 16	7, 8
*F*	21.04	9.20	52.94	74.56	97.02	381.05
*p*	<0.0001	<0.0001	<0.0001	<0.0001	<0.0001	<0.0001

**Table 2:** One-way ANOVA statistics for each year with population as a single factor.

**2018**	**June-20**	**June-28**	**July-2**	**July-5**	**July-10**	**July-15**	**July-21**	**July-27**
*F*_2,4_	0.85	0.92	1.43	0.35	9.04	18.83	25.47	71.40
*p*	0.4921	0.4676	0.3403	0.7233	0.0328	0.0092	0.0053	0.0007
**2019**	**June-21**	**June-26**	**July-1**	**July-7**	**July-12**	**July-17**	**July-23**	**July-26**
*F*_2,5_	1.47	3.79	19.40	90.82	6.80	20.00	52.12	13.03
*p*	0.3156	0.0997	0.0044	0.0001	0.0375	0.0041	0.0004	0.0104

## Data Availability

Data is contained within the article and Supplementary Material.

## Supplementary Materials

The following are available online at https://www.mdpi.com/2075-4450/12/3/232/s1, Figure S1: Schematic diagram of an open top chamber for maize plant, Figure S2: Photo of an open top chambers (**left**) and field screen cages (**right**), Figure S3: Daylength in Gongzhuling from June 20 to September 30. Table S1: The number of *Ostrinia furnacalis* adults collected in Harbin, Table S2: The number of *Ostrinia furnacalis* moths collected in Gongzhuling.
