# Supplementary material for: Voltine Ecotypes of the Asian Corn Borer and Their Response to Climate Warming
Source: Insects. 2021 Mar 9;12(3):232. doi: 10.3390/insects12030232 (PMC7998394; doi:10.3390/insects12030232)
Supplement: Supplementary file 1 [file insects-12-00232-s001.pdf]

Table S1. The number of *Ostrinia furnacalis* adults collected in Harbin.

Time Period	2017			2018			2019		
	Collection Date	No. of Females	No. of Males	Collection Date	No. of Females	No. of Males	Collection Date	No. of Females	No. of Males
I	Jun. 13–15	238	156	Jun. 14–16	264	216	Jun. 17, 18	135	100
II	Jun. 19, 20, 22	272	208	Jun. 19, 21, 22	285	171	Jun. 22, 23	155	115
III	Jun. 23–25	334	160	Jun. 25, 26, 28	284	116	Jun. 27, 28	130	75
IV	Jun. 28, 29; Jul. 2	200	167	Jun 29, 30	286	149	Jul. 2–4	145	115
V	Jul. 3–5	302	173	Jul. 4–6	333	137	Jul.8, 9	200	120
VI	Jul 8, 9	212	138	Jul. 9–11	312	188	Jul. 13, 14	150	110
VII	Jul. 13–15	267	143	Jul. 15–17	186	119	Jul. 18, 19	120	80
VIII	Jul 18, 19, 21	229	61	Jul. 20–22	210	158	Jul. 22, 23	140	115

Table S2. The number of *Ostrinia furnacalis* moths collected in Gongzhuling.

Time Period	2018			2019		
	Collection Date	No. of Females	No. of Males	Collection Date	No. of Females	No. of Males
I	Jun. 15–16	50	35	Jun. 19	45	25
II	Jun. 19–20	60	40	Jun. 23	60	40
III	Jun. 25–26	40	45	Jun. 27-28	45	40
IV	Jun. 29	50	30	Jul. 2-3	50	50
V	Jul. 5	45	25	Jul. 7	55	45
VI	Jul. 9–10	60	45	Jul. 15–16	45	20
VII	Jul. 14–15	40	35	Jul. 20	60	30
VIII	Jul. 21–22	45	45	Jul. 24	50	20

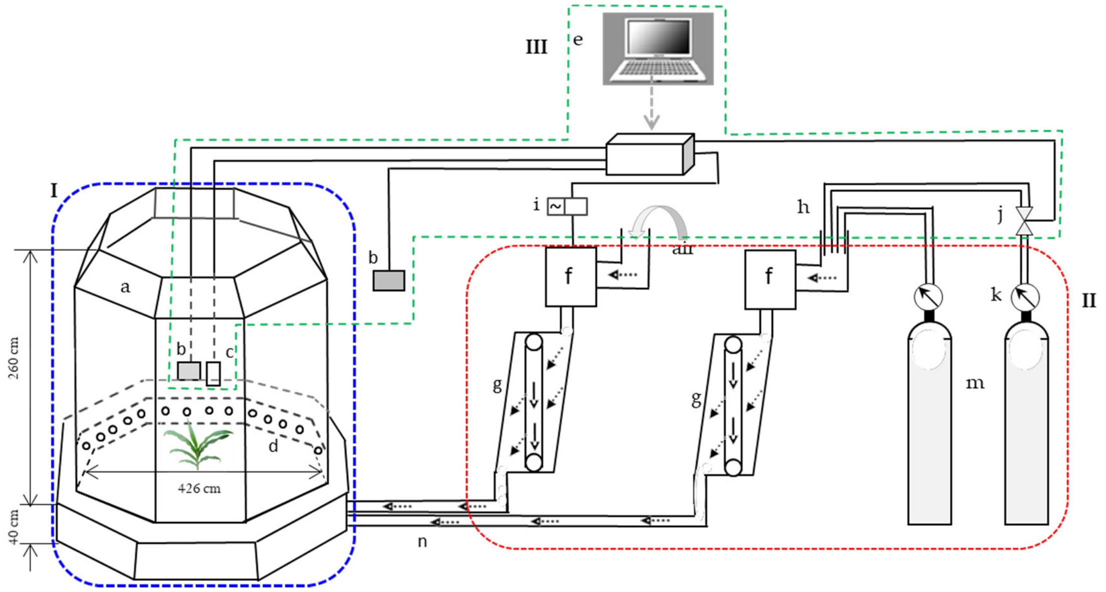

Figure S1. Schematic diagram of an open top chamber for maize plant. (**a**) glass chamber; (**b**) temperature and humidity sensor (placed in louver) (THP-T26-V5, Tongdy sensing Technology Corporation, Beijing, China); (**c**) CO_2 sensor (TGP108-V5, Tongdy sensing Technology Corporation, Beijing, China); (**d**) air inlets; (**e**) digital control system (CO_2 sensor, a chip core, computer and software package); (**f**) air fans; (**g**) cooling system; (**h**) CO_2 inlet; (**j**) magnetic valve; (**k**) CO_2 pressure reducer; (**m**) CO_2 tank; (**n**) pipe; ◄···· air flow direction.

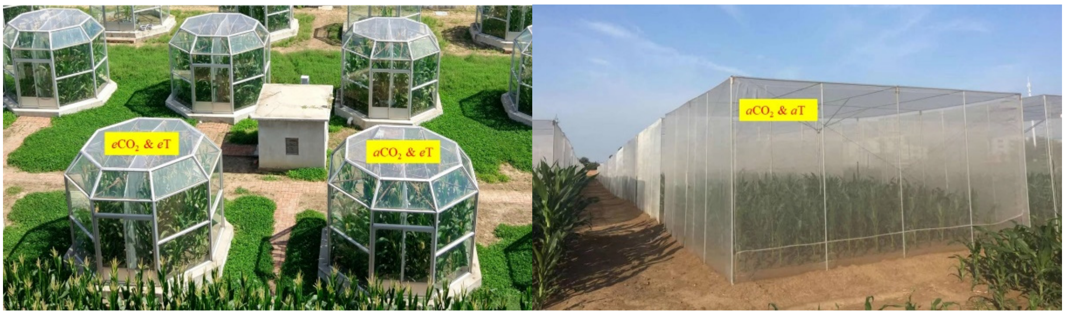

Figure S2. Photo of an open top chambers (**left**) and field screen cages (**right**).

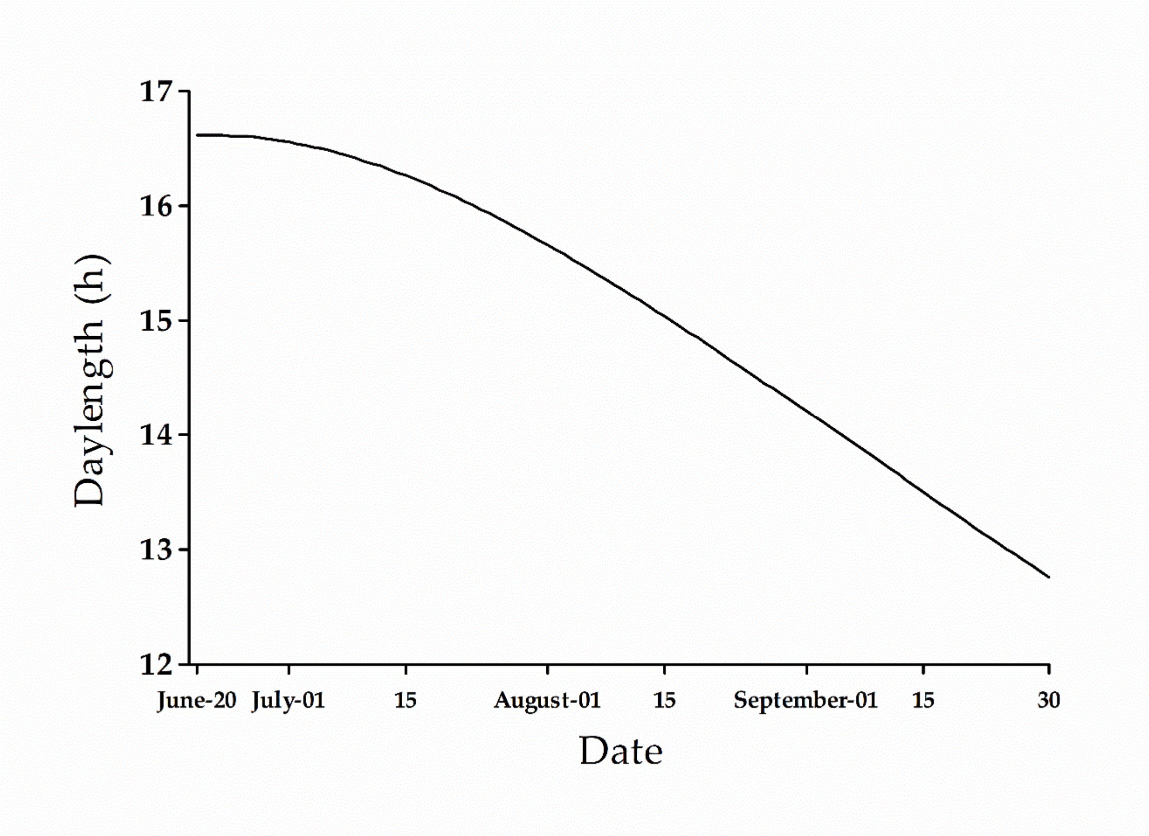

Figure S3. Daylength in Gongzhuling from June 20 to September 30.
